# Host Plant Volatiles and the Sexual Reproduction of the Potato Aphid, *Macrosiphum euphorbiae*

**DOI:** 10.3390/insects5040783

**Published:** 2014-10-24

**Authors:** Jessica Hurley, Hiroyuki Takemoto, Junji Takabayashi, Jeremy N. McNeil

**Affiliations:** 1 Department of Biology, Western University, London, ON, N6A 5B7, Canada; E-Mail: jessica.hurley@basf.com; 2 Center for Ecological Research, Kyoto University 2-509-3, Hirano, Otsu 520-2113, Japan; E-Mails: uhtakem@ipc.shizuoka.ac.jp (H.T.); junji@ecology.kyoto-u.ac.jp (J.T.); 3 Research Institute of Green Science and Technology, Shizuoka University 836, Ohya, Shizuoka 422-8529, Japan

**Keywords:** potato aphid, *Macrosiphum euphorbiae*, sexual morphs, mate location, female sex pheromone, host plant volatiles

## Abstract

In late summer, heteroecious aphids, such as the potato aphid, *Macrosiphum euphorbiae*, move from their secondary summer host plants to primary host plants, where the sexual oviparae mate and lay diapausing eggs. We tested the hypothesis that volatiles of the primary host, *Rosa rugosa*, would attract the gynoparae, the parthenogenetic alate morph that produce oviparae, as well as the alate males foraging for suitable mates. In wind tunnel assays, both gynoparae and males oriented towards and reached rose cuttings significantly more often than other odour sources, including potato, a major secondary host. The response of males was as high to rose cuttings alone as to potato with a calling virgin oviparous female. These findings are discussed within the seasonal ecology of host alternating aphids.

## 1. Introduction

Host plant volatiles are one of the important cues used by females of many insect species to locate suitable oviposition sites [1,2]. Moreover, plant volatiles may be important in the onset of sexual maturation and/or the emission of sex pheromones by virgin females [3,4]. Furthermore, males may use plant volatiles when searching for mates and show higher levels of response to the combination of plant volatiles with the sex pheromone, than to the female pheromone alone [5,6,7,8,9,10].

In temperate regions, aphids reproduce asexually during the summer, but in response to decreasing day length and temperature, as well as declining plant quality [11], they produce sexually reproducing males and females in the fall. Monecious species remain on one host species throughout the year, while heteroecious (host alternating) species move between primary overwintering and secondary summer host plant species. In the fall, parthenogenetic females of heteroecious species produce gynoparae (alate parthenogenetic females) and alate males on the secondary summer hosts, and these morphs migrate to the primary overwintering hosts. Upon arrival on a suitable primary host, the gynoparae produce sexual apterous oviparae, who emit sex pheromones from scent plaques on their hind tibiae to attract sexually mature alate males [11,12,13,14], and once mated, produce eggs that remain in diapause throughout the winter [12].

Host-alternating species are often highly specialized with respect to both their primary and secondary hosts [11,14], and it has been proposed that volatiles may play a vital role in the location of primary host plants by both gynoparae and males [5,6]. A positive response by gynoparae would ensure that their progeny, the oviparae, develop on suitable host plants. In the case of foraging males, responding to primary host plant volatiles alone, or combined with the female sex pheromone, would increase the probability of finding habitats where there are oviparae [15,16,17,18]. However, there has not been a great deal of experimental work examining the responses of either gynoparae or males, and the results available are quite variable. While the gynoparae of *Rhopalosiphum padi* [16] and *Phorodon humuli* [6,19] responded positively to volatiles from their primary host plants, the gynoparae of *Aphis fabae* did not [20]. Similarly, while *R. padi* [16] males were attracted to the volatiles of their primary host plants, those of *P. humuli* [19], *Cryptomyzus galeopsidis* [5] and *Sitobion fragariae* [21] were not.

In this study, we undertook studies to determine if volatiles from *Rosa rugosa*, a primary host plant of the potato aphid, *Macrosiphum euphorbiae*, are attractive to the gynoparae and males. We predicted that if primary host plant volatiles were important in the location of overwintering sites, then both morphs would be more responsive to, and take less time to reach, a primary host plant than a summer secondary host. In the case of males, we also tested their responses to primary and secondary host plant volatiles in the presence and absence of a calling virgin oviparous female, predicting that while primary host plant volatiles alone would be attractive to males, responses would increase when both olfactory cues were presented simultaneously.

## 2. Experimental Section

### 2.1. Insects

*Macrosiphum euphorbiae* used in these experiments came from a laboratory colony established from potato fields near Quebec City. The aphids were maintained on potato seedlings, *Solanum tuberosum* c.v. Norland, at 21 ± 1 °C, 60% ± 10% RH under a 16L:8D photoperiodic regime.

Sexual morphs were obtained by rearing parthenogenetic apterous individuals at 18 ± 1 °C, 60% ± 10% RH under a 10L:14D photoperiodic regime (as described in [22]). Late instar nymphs were sexed based on morphology and reared separately to ensure that the oviparous females were virgins and that males had not been exposed to the sex pheromone prior to being assayed.

### 2.2. Bioassays

Bioassays were performed in a laminar airflow wind tunnel (140.8 cm long × 64.8 cm wide × 64.8 cm high) located in an environmental chamber maintained at 21 ± 1 °C and 60% ± 10% RH. The tunnel was illuminated by two banks of overhead daylight fluorescent lights. The insects being tested, at a wind speed between 0.4–0.6 m/s, were released from a platform 10 cm downwind of the platform with the odour source. The two platforms were connected by a string bridge, as previous experiments on this species found that males will more frequently walk rather than fly towards an odour source [22]. In the case of both gynoparae and males, there were four replicates (12 individuals/replicate) for each odour source. Each individual was tested for 120 s and was considered unreceptive if no behavioral changes were seen in this time period. For each odour, we recorded the proportions of individuals that oriented to and reached the source, as well as the times taken to exhibit these behaviors. In addition, we noted whether individuals walked or took flight. Individuals were only tested once.

The response of 1–3-day-old gynoparae was tested when exposed to a 10-cm cutting (leaves only) of *Rosa rugosa* (a primary host), *Solanum tuberosum (*a secondary host) or *Buddleia* sp*.*, (a non-host often found in close proximity to roses in the region), as well as to a 10-cm glass rod to provide a clean air control. The potato plants and rose bushes were grown in a greenhouse at 24 ± 1 °C under natural light conditions, while butterfly bush cuttings were taken from bushes growing in a garden next to the greenhouse. The stem of each cutting (and the glass rod) was inserted through the lid of a 20-mL transparent plastic containing water. Cuttings were replaced for each replicate.

In the case of 1–3-day-old virgin males, we tested their response to 10-cm cuttings of either *R. rugosa* or *S. tuberosum* alone or cuttings with a 6–8-day-old calling virgin female. The oviparae were transferred to the plants at least 60 min before the experiments were carried out, and assays were only conducted if the females were actively calling, as evidenced by the raised position of the hind tibiae [23]. Again, containers with a glass rod were used as clean air controls.

### 2.3. Statistics

Prior to analysis, the proportions of either the 12 gynoparae or males that oriented and reached the source in each replicate (N = 4) were normalized using an arcsine square root transformation, while the times taken for responding individuals that reached the source in all replicates was log transformed to assure the homogeneity and homoscedasticity of the data. The proportions of gynoparae in each replicate (N = 4) orienting to and reaching the source were analysed using one-way ANOVAs (plant source), while data for males were analysed using two-way ANOVAs (plant source and presence or absence of a calling female). In the case of males, it was an incomplete factorial design, as females would not call when placed on the glass rods. When there were significant factors, the ANOVAs were followed by Tukey’s HSD tests using SPSS 2006.

## 3. Results

**Figure 1:**
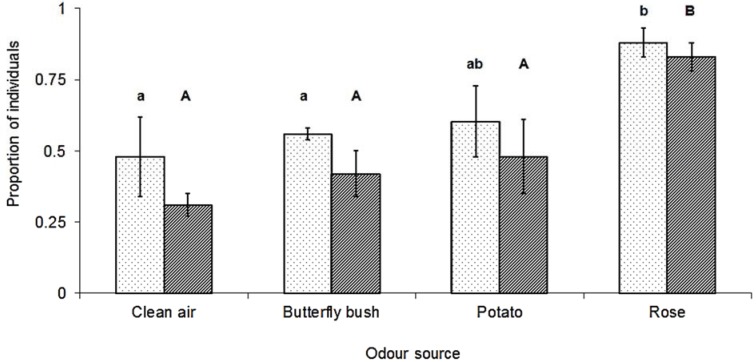
Proportion (X ± SEM) of *M. euphorbiae* gynoparae (N = 48 in each treatment) that oriented towards (dotted bars) or reached (striped bars) the different odour sources in wind tunnel assays. Columns with different letters, either for orienting to or reaching the source, are significantly different (Tukey’s HSD, *p* < 0.05).

**Figure 2:**
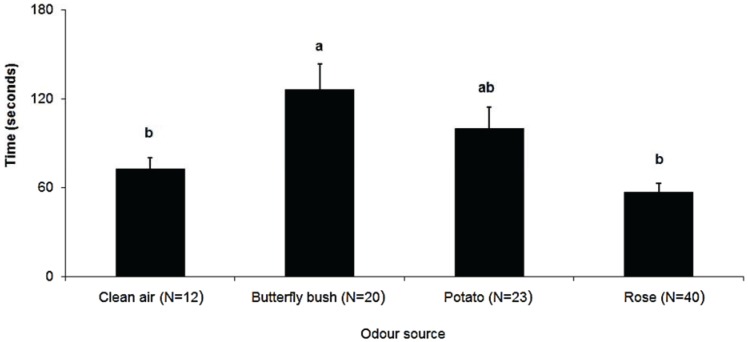
Mean time (X ± SEM) taken by *M. euphorbiae* gynoparae to reach different odour sources in wind tunnel assays (N = number of responding males). Columns with different letters are significantly different (Tukey’s HSD, *p* < 0.05).

The proportion of *M. euphorbiae* gynoparae that oriented to (F_(3,12)_ = 4.97, *p* = 0.018) and reached (F_(3,12)_ = 7.87, *p* = 0.004) the different sources differed significantly (Figure 1), with the highest responses being to the primary host plant. Furthermore, the origin of the odour source significantly affected the time taken for gynoparae to reach the source (F_(3,12)_ = 7.61, *p* < 0.001, due in large part to the increased time to reach the butterfly bush cuttings (Figure 2).

**Figure 3:**
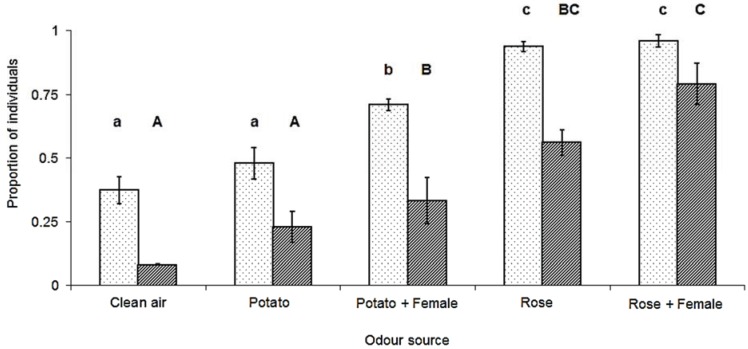
Proportion (X ± SEM) of *M. euphorbiae* males (N = 48 in each treatment) that oriented towards (dotted bars) or reached (striped bars) the different odour sources in wind tunnel assays. Columns with different letters, either for orienting to or reaching the source, are significantly different (Tukey’s HSD, *p* < 0.05).

**Figure 4:**
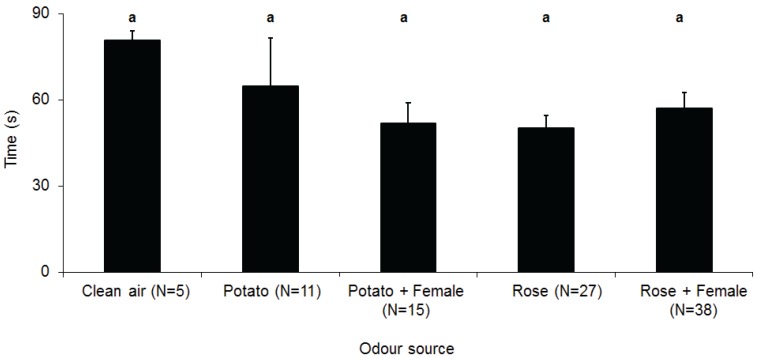
Mean time (X ± SEM) taken by *M. euphorbiae* males to reach different odour sources in wind tunnel assays (N = number of responding males). Columns with different letters are significantly different (Tukey’s HSD, *p* < 0.05).

As with gynoparae, the proportion of *M. euphorbiae* males orienting to and reaching the source varied significantly depending on the odour source (Figure 3). There was a significant effect of both the plant (F_(2,15)_ = 43.35, *p* < 0.001) and the calling female (F_(1,15)_ = 5.9, *p* = 0.028) on the proportion of males orienting to the source, but there was no significant plant by female interaction (F_(1,15)_ = 1.66, *p* = 0.217). Significantly more males oriented to the rose, regardless of whether a calling female was present or not, than to all other sources, while more males oriented towards a potato plant with a calling female than to potato alone or to the clean air control. There was also a significant effect of both the plant (F_(2,15)_ = 19.20, *p* < 0.001) and the presence of a calling female (F_(1,15)_ = 5.88, *p* = 0.028) on the proportion of males reaching the source, but again, there was no significant plant by female interaction (F_(1,15)_ = 1.12, *p* = 0.306). While more males reached the combination of calling female and rose, this was not significantly different than rose alone. Similarly, the number of males reaching the rose cutting alone was not significantly different from the potato cutting with a calling female, but was significantly more attractive than potato or clean air alone (F_(2,15)_ = 27.97, *p* < 0.001). However, for responding males, the time taken to reach any source was not significantly affected by the plant source (F_(2,15)_ = 0.017, *p* = 0.984) or the presence or absence of a calling female (F_(2,15)_ = 0.11, *p* = 0.741) (Figure 4).

## 4. Discussion

Aphids are weak flying insects, with both the speed and direction of flight being determined by prevailing wind conditions [24], and it has been estimated that approximately 99% of alates do not locate suitable hosts [25]. However, they do have control over landing behaviour [14] and potentially make landing decisions based on visual and chemical information [26,27,28]. Therefore, a positive response of both gynoparae and males to primary host plant volatiles, carried aloft by thermals during the diurnal flight period of aphids, could certainly increase the probability of reproductive success.

*Macrosiphum euphorbiae* gynoparae, like those of some other aphid species [6,19], showed a significantly higher response to volatiles from the preferred primary host plant over the other odour sources tested, supporting the hypothesis that this species uses volatile cues from primary hosts to locate suitable hosts for the subsequent deposition of oviparae. These volatiles are probably used in combination with visual cues, such as plant reflected wavelengths that modulate aphid landing behaviour [26,27,28], in host plant selection. Upon landing, the behaviours associated with settling behaviour would ensure that the antennal sensilla, as well as those on the proboscis, would detect close-range chemical stimuli associated with host plant recognition [13,28,29]. Allelochemicals from suitable primary host plants may induce the onset of reproduction and/or increase the reproduction output of gynoparae [30]. However, in the case of *M. euphorbiae* gynoparae, there were no significant differences in their total nymph output, whether maintained on primary or secondary hosts [31].

Male *M. euphorbiae* also showed a positive response to primary host plant volatiles, and there are a number of reasons to believe that these olfactory cues are of considerable significance in long‑distance mate location. First, given the relative size of the oviparae and the host plant, it is not unreasonable to assume that the intensity of the chemical signals emitted from host plants would be significantly greater that those from a receptive female. Thus, compared with the female sex pheromone, the host plant volatiles would serve as longer distance cues akin to “host habitat location” prior to “host location” proposed by Vinson for foraging parasitoids [32]. Furthermore, in both laboratory and field assays, *M. euphorbiae* males generally walked rather than flew to a calling female [22], so it is unlikely that weak flying male aphids are capable of strong directed upwind flight to a pheromone source, as reported for larger, strong flying insects, like moths and beetles [33,34,35,36]. Thus, the female sex pheromone probably serves as an important cue once the males have located the host plant. Furthermore, the sex pheromones of the majority of species studied to date [5,6,15,37,38,39,40] are primarily made up of the same two monoterpenoids (nepetalactol and nepetalactone) and, thus, may not provide reliable species-specific cues. It was suggested that species specificity could be obtained by the use of differing ratios of the two components [37], but given that (i) ratios may change with female age [15,37,39], (ii) males respond to quite a wide range of ratios in laboratory assays [22,38] and (iii) males of several aphid species can be trapped using a single pheromone ratio [3], it would appear that ratios alone would not provide an effective means of locating suitable mates or for reproductive isolation. Thus, the volatiles from the specific primary host plant in combination with the sex pheromone may increase the ability of males to discriminate between a conspecific and heterospecific sex pheromone [5,28]. Recently, Pope *et al.* [10] provided convincing evidence that the combination of host plant volatiles and female sex pheromones could play an integral role in the reproductive isolation of aphid species that use similar primary host plants. The responses observed with *M. euphorbiae* show that males are clearly attracted to the primary host plant volatiles over those from other host plants within the system, although the response to rose volatiles in combination with the sex pheromone from a calling female was not significantly greater than to the rose volatiles alone. However, as *M. euphorbiae* males respond to a wide range of sex pheromone ratios [15], it would be of interest to determine if the breadth of the response window is reduced in the presence of the primary host volatiles.

## 5. Conclusions

Our study looked at the responses of *M. euphorbiae* gynoparae and males over short distances in a wind tunnel, so the responsiveness of both morphs to the different chemical cues tested in our assays needs to be investigated over a range of distances under field conditions. If our laboratory finding are supported under field conditions, then it would seem that the most effective management tool for aphid pests would be the use of primary host plant volatiles alone, or in combination with the sex pheromones. This could be achieved through the strategic planting of specific plants to attract or deter aphids [19] or the deployment of synthetic lures once the specific volatiles from primary hosts have been identified. The objective would be to decrease pesticide applications, thereby reducing the negative environmental effects of pesticide use and the development of pesticide-resistant aphid strains [41].
